# A Study of a New Certified Reference Material for Accurate Determination of the Main *Fusarium* Mycotoxins in Whole-Wheat Flour

**DOI:** 10.3390/foods12234358

**Published:** 2023-12-02

**Authors:** Li Li, Peng Li, Yu Wu, Jin Ye, Zongwang Li, Songxue Wang

**Affiliations:** 1 Academy of National Food and Strategic Reserves Administration, Beijing 100037, China; ll@ags.ac.cn (L.L.); lp@ags.ac.cn (P.L.); wyu@ags.ac.cn (Y.W.); yj@ags.ac.cn (J.Y.); lzw18735877886@163.com (Z.L.); 2 School of Health Science and Engineering, University of Shanghai for Science and Technology, Shanghai 200093, China

**Keywords:** certified reference material, *Fusarium* mycotoxins in whole-wheat flour, ID-LC/MS/MS, characterization, homogeneity and stability study, uncertainty evaluation

## Abstract

Matrix certified reference materials (CRMs) play a critical role in analytical method validation and the assurance of reliable measurement results. A certified reference material (GBW(E)100813) for whole-wheat flour was developed to ensure an accurate and reliable measurement of the main *Fusarium* mycotoxins (deoxynivalenol (DON), nivalenol (NIV), deoxynivalenol-3-glucoside (DON-3G), and zearalenone (ZEN)). CRM candidates were prepared using sun-drying, grinding, sieving, homogenising, packaging, and gamma irradiation. The final produced CRM was packaged at 50 g per unit and stored at 20 °C. Certification was performed using isotope dilution-liquid chromatography–tandem mass spectrometry. CRM characterization was performed in eight laboratories in accordance with the requirements of ISO Guide 35. The certified values and expanded uncertainties (at a confidence of 95%, k = 2) for DON, NIV, DON-3G, and ZEN were determined to be 0.98 ± 0.12 mg/kg, 1.37 ± 0.20 mg/kg, 242 ± 35 μg/g, and 382 ± 50 μg/g. The CRM was sufficiently homogeneous between and within bottles, and remained stable for up to 12 months at 20 °C and 9 days below 40 °C for transportation. Thus, CRM can be used for quality control and method validation to ensure the accurate and reliable quantification of the main *Fusarium* mycotoxins in whole-wheat flour.

## 1. Introduction

Whole-wheat flour has attracted increasing attention globally as a novel form of wheat. However, wheat can be infested with different species of *Fusarium* during crop growth, harvest, transportation, storage, and processing, which not only affects wheat quality (e.g., germination rate or nutrient content), but is also a serious problem due to the accumulation of *Fusarium* mycotoxins, which affect the safety of humans and livestock [1,2,3]. The two main classes of *Fusarium* mycotoxins–monoterpenoids and zearalenones, directly affect wheat-based products because they are not broken down during food processing [4]. There are two types of trichothecenes, type A and type B. Relevant to human health, type B trichothecenes include deoxynivalenol (DON), 3- and 15-acetyl deoxynivalenol (3-ADON and 15-A-DON), nivalenol (NIV), and fusarenon-X (FX), of which DON, FX, and NIV are the most dominant and common naturally occurring contaminants of cereals in the *Fusarium* mycotoxins family [5,6,7,8]. These B-type monosporins readily bind to eukaryotic ribosomes, causing strong cytotoxicity in eukaryotic cells, inhibiting translation, and causing severe damage to the organism [9,10]. This can additionally cause anorexia, diarrhoea, reduced weight gain, haematological disorders, and immunosuppression [11]. Deoxynivalenol-3-glucoside (DON-3G), the most masked DON mycotoxin, is a modified/conjugated compound produced in plants through glycosylation reactions to protect them from foreign organisms [12,13]. DON-3G is considered less harmful than its parent form. However, research has indicated that DON-3G can transform into DON during human and animal intestinal digestion [14,15]. The European Commission (EC) holds that DON-3G is as toxic as DON [6]. Therefore, it is essential to routinely analyse DON-3G in cereals and related products to avoid underestimation of the total DON level. Zearalenone (ZEN) is an oestrogenic mycotoxin with strong teratogenic effects and reproductive toxicity [16]. DON and ZEN have been shown to synergistically affect brain function in mice through synergistic enhancement [17]. In addition, *Fusarium* is mainly found in the outer layer of wheat grains, which may contain higher levels of mycotoxins than refined flour, which is made from whole grains without the removal of the epidermal part [15,18].

In recent years, wheat and its products have become susceptible to simultaneous contamination by several *Fusarium* mycotoxins, owing to factors such as climatic anomalies and changes in farming practices; therefore, it is essential to analyse and monitor *Fusarium* mycotoxins in cereals and related products. Such analysis involves complex sample pretreatment and highly selective instrumental analysis; therefore, quality control is required. Validation of the performance of mycotoxin analytical methods is essential to ensure the reliability of the analytical results. As reported in several studies [19,20,21,22,23], the use of a certified reference material (CRM) is essential to validate analytical methods. The addition of compounds to matrix samples for test recovery (spiking) has been widely used in many testing laboratories to evaluate analytical methods. In addition, the supplemental use of CRMs is helpful because the conditions of the analytes in CRMs are like those in actual samples. In addition, CRMs are essential for developing new analytical methods and achieving metrological traceability, as well as for proficiency testing and adherence to internationally agreed standards. Additionally, with the development of MS technology, there is a tendency to use one method for the simultaneous detection of multiple mycotoxins, which requires matrix standards of multiple mycotoxins to assess method performance. Although several fungal toxin standards (CRMs) have been developed, CRMs for the multiple *Fusarium* mycotoxins in food matrices are scarce. No reference materials have been reported for DON, ZEN, or NIV nor for DON-3G in whole-wheat flour.

To address this gap, our laboratory initiated a collaborative project to develop reference materials for DON, DON-3G, NIV, and ZEN in whole-wheat flour. Combined with our contamination monitoring study in the main wheat-producing areas [24], wheat naturally contaminated with *Fusarium* mycotoxins was chosen as a candidate material. The candidate material, obtained by natural drying, homogenisation, and sieving, was optimised and confirmed by isotope dilution liquid chromatography–tandem mass spectrometry (ID-LC-MS/MS) based on a laboratory-established method for the detection of 16 fungal toxins in grains, which was identified as the reference method for CRM [25,26]. The homogeneity and stability of reference substances were monitored. The uncertainties evaluation introduced during its development were assessed, and six laboratories that have extensive experience in the detection of mycotoxins using various tests were invited to participate in an interlaboratory comparison study to certify the CRM candidate materials based on the measurement results. The State Administration for Market Regulation in China approved and numbered this matrix-certified reference material as GBW(E) 100816.

## 2. Materials and Methods

### 2.1. Reagents, Standards, and Materials

The CRM for ZEN in methanol (GBW(E)100301, 20.52 ± 0.25 μg/mL) and DON in acetonitrile (GBW(E)100304, 100.7 ± 3.5 μg/mL) were developed by Academy of National Food and Strategic Reserves Administration (Beijing, China); the standards for NIV in acetonitrile and DON-3G in acetonitrile were purchased from Pribolab (Pribolab Biotech Co., Ltd., Qingdao, China). These standards were used as the primary reference material for DON, ZEN, DON-3G, and NIV for the certification of whole-wheat flour CRM. The stable isotope ^13^C_15_-DON (25.5 μg/mL), ^13^C_15_-NIV (25.5 μg/mL), ^13^C_21_-DON-3G (10.4 μg/mL), and ^13^C_18_-ZON (25.0 μg/mL) used in ID-LC-MS/MS (in acetonitrile) were also purchased from Pribolab (Pribolab Biotech Co., Ltd., Qingdao, China). HPLC-grade organic solvents (acetonitrile and methanol) were purchased from Thermo Fisher Scientific (Carlsbad, CA, USA). Formic acid, ammonium acetate, and acetic acid were obtained from J&K Amethyst Chemicals (Sinopharm Chemical Reagent Co., Ltd., Shanghai, China). Milli-Q Q-Gard1 monolithic water purification system (Merck, Germany) was used to prepare ultrapure water.

### 2.2. Selection and Preparation of the Candidate CRM

Their occurrence rates and contamination levels of *Fusarium* mycotoxins in wheat were reviewed from the “Mycotoxin Monitoring Database in Wheat in Main Production Areas of China” developed by ASAG. Among those, wheat showed the occurrence of *Fusarium* mycotoxins such as DON, NIV, DON-3G, ZEN, 3-ADON, and 15-ADON in China at higher levels, which were consistent with other authors’ studies. About 100 kg of wheat were collected from different administrative regions of Hubei and Anhui provinces and screened for target *Fusarium* mycotoxins using the LC-MS/MS method and preciously published, of which about 30 kg were used as candidate raw materials. The wheat pellets were dried naturally and then ground using a Lecher SR300 grinder (Haan, Germany). The whole-wheat flour was then sieved using a XZS1200 vibrating sieve (Henan, China) and passed through a 0.5 mm screen. After that, the material was homogenized by a ZKH V-mixer (Jiangsu, China) for 36 h and packed in aluminium foil bags at 50 g per unit using an automatic dispenser (Tianjin, China) and vacuum sealed using a DZ-980 vacuum packaging machine (Beijing, China). In total, 515 packaging units were prepared with batch number 2A10. Each unit was irradiated and sterilized by Co60 (irradiation dose of 10 kGy) and kept in a cool and dry environment.

### 2.3. Preparation of Standard Solutions

A certain amount of ^13^C1_5_-DON, ^13^C_15_-NIV, ^13^C_21_-DON-3G, and^13^C_18_-ZEN were accurately pipetted into a 10 mL volumetric flask fixed the volume to the scale with dilution solution (acetonitrile: acetic acid: water = 35:0.5:24.5). The mixed internal standard solution was examined by LC-MS/MS and the corresponding target mycotoxins were not detected, which did not affect the quantification of CRM. A certain amount of DON, NIV, DON-3G, and ZEN were accurately pipetted into a 10 mL volumetric flask and the volume was fixed to the scale with the dilution solution (acetonitrile: acetic acid: water = 35:0.5:14.5). The prepared solutions were sealed in brown ampoule bottles and stored under −18 °C. Single point calibration was used, and the calibration standard was freshly prepared by using the working solution with closing to the concentration level of the target *Fusarium* toxin in CRM candidates and spiked with internal solution to make a 1:1 isotope ratio.

### 2.4. Particle Size Distribution

The HELOS-OASIS was equipped with a vibrating chute funnel feeder, and the sampling method was pneumatic suction. One gram of whole-wheat flour was weighed into the sample stage, and the instrument’s test range was set then tested.

### 2.5. Characterization

#### 2.5.1. Sample Pretreatment

Five grams of whole-wheat flour were weighed into a 50 mL screw-cap centrifuge tube. Twenty milliliters of MeCN-water-acetic acid extraction solvent (70 + 29 + 1, *v*/*v*/*v*) were added and shaken on a vortex shaker for 1 min. This was followed by bouncing on a rotary shaker for 30 min and sequential centrifugation (3500× *g*, 5 min). The supernatant (0.5 mL) was aspirated into a 1.5 mL centrifuge tube, diluted with 0.5 mL water, and shaken on a vortex shaker for 1 min, followed by centrifugation (7200× *g*, 10 min, 4 °C). The supernatant was filtered through a 0.22 µm PTFE filter. An aliquot (180 µL) of the filtrate was added, with 20 µL SID working solution, into the 400 µL micro inserts in the injection vials. The tubes were capped, mixed, and injected into the LC-MS/MS system.

#### 2.5.2. ID-LC/MS/MS Measurement

**Figure 1:**
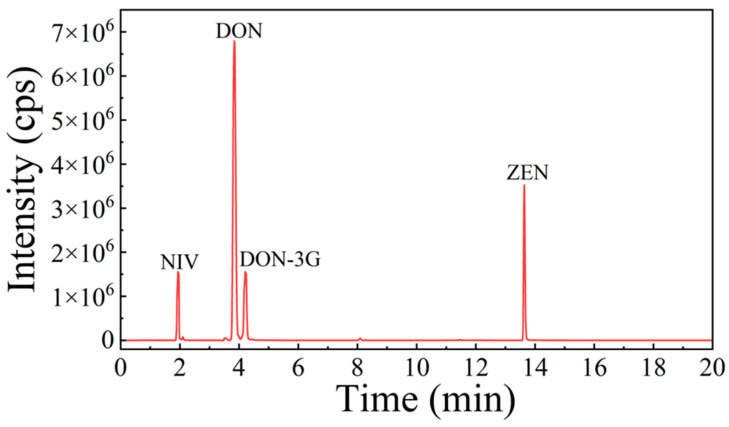
TIC chromatograms of four *Fusarium* mycotoxin in whole-wheat flour.

Chromatographic separation was realized using the QTRAP^®^ 6500+ LC-MS/MS system (SCIEX, Framingham, MA, USA) with a CORTECS UHPLC C18 column (100 mm × 2.1 mm, 1.6 μm; Waters, Milford, MA, USA). The injection volume was 2 μL, and the flow rate was 0.3 mL/min with a column temperature of 40 °C. The mobile phases were water with 0.1% formic acid and 1 mM ammonium acetate (A) and MeOH (B). The gradient elution program was linearly applied by changing the mobile phase composition as follows: B 10–10%, 0–2 min; B 20–21%, 2–3 min; B 21–26%, 3–4 min; B 21–26%, 4–5 min; B 26–26%, 5–7 min; B 26–60%, 7–10.5 min; B 60–60%, 10.5–13.5 min; B 60–95%, 13.5–14.5 min; B 95–95%, 14.5–17 min; B 95–10%, 17–18 min; B 10–10%, 18–21 min. Samples were analysed in the MRM, and the ion source temperature and ion spray voltage under ESI (±) were set to 450 °C and ±4.5 kV, respectively. Nitrogen gas was applied as the cone gas (GS1), curtain gas, heating gas (GS2), and collision gas at pressures of 35, 30, 25, and 10 psi, respectively. MS/MS conditions of DON, NIV, DON-3G, ZEN were shown in Table 1. A chromatogram of four Fusarium mycotoxin in whole-wheat flour was shown in Figure 1.

Quantification of *Fusarium* toxin in CRM was performed using a single-point isotope calibration method, as described in the Formula (1).

(1) $$C_{sample}=\frac{CR_{std}\times AR_{sample}\times C_{IS}}{m_{sample}\times AR_{std}}\times f$$

where $C_{sample}$ is the mass fraction of the target mycotoxin in CRM of whole-wheat flour; $AR_{sample}$ and $AR_{std}$ is the ratio of the sample extraction detection solution and the area of the corresponding isotope standard solution, respectively; $CR_{std}$ is the ratio of the c solution and the mass of the corresponding isotope standard solution, for the convenience of calculation isotope standard solution mass is 1 μg by default; and $m_{sample}$ is the mass of the sample used for analysis and is f the dilution multiple, 8.

### 2.6. Homogeneity Testing

According to the stratified random sampling scheme, 15 packaging units were randomly sampled from the batch for the homogeneity testing of DON, NIV, DON-3G, and ZEN in whole-wheat flour CRM candidates. Mass fractions of four *Fusarium* mycotoxins of three subsamples were selected from each bag, top, middle, and bottom, and analysed using ID-LC/MS/MS. Meanwhile, to reduce the effect of instrument orientation drift (mainly referring to the unavoidable drift error allowed by the assay) caused by the measurement time on the statistical results, three determinations were performed on all treated samples. The determination of all treated samples was carried out in the order of the assay first, then in reverse order, and finally in odd–even order. According to the JJF 1343-2012 [27], the measurement results were statistically analysed by using one-way analysis of variance (ANOVA) at a 95% confidence level for within/between bottle homogeneity. The uncertainty components introduced by the inhomogeneity were calculated and included in the total uncertainty of the standards.

### 2.7. Stability Assessment

The long-term stability of the matrix CRMs at 20 °C storage condition was assessed for 0, 1, 4, 8, and 12 months. For short-term stability, the stability was studied at 40 °C for 1, 3, 5, 7, and 9 days were investigated. Two units were randomly selected and measured in triplicates at predetermined times. All samples were measured using ID-LC-MS/MS, and the stability was assessed using regression analysis.

### 2.8. Characterization and Uncertainty Evaluation

Based on the requirements of JJF 1006-1994, the characterization of four *Fusarium* mycotoxins in whole-wheat flour was carried out for quantitative value determination. Six laboratories with experience in mycotoxin detection were selected for testing, including Beijing Agricultural Quality Standards and Testing Technology Research Center (Beijing, China), Scientific Research Institute of the State Administration of Grain and Material Reserves (Beijing, China), Rome Laboratory Testing Service (Wuxi, China), Guangxi Zhuang Autonomous Region Grain and Oil Quality Inspection Center (Nanning, Guangxi), National Food Safety Risk Assessment Center (Beijing, China), and Zhejiang Tsinghua Yangtze River Delta Research Institute (Jiaxing, China). CRM candidate samples of three units, as well as the standard solutions and isotope internal standard solution were provided to each participating laboratory. All laboratories were required to use the same ID-LC-MS/MS method as a reference method for two units and three subsamples for each unit. This study used single point calibration to calculate mycotoxins in whole-wheat flour. Meanwhile, GBW(E)100382 and GBW(E)100384 were selected for quality control of DON and ZON in whole-wheat flour, respectively, and NIV and DONG-3G were quality controlled by spiked recovery near the quantitative values to ensure quality control of the experimental process and valid determination results.

The value of the candidate whole-wheat flour standard was determined as the total average of the results of several laboratories, and the specific calculation formula is shown in (2).

(2) $$\overset{=}{x}=\frac{\sum_{i=1}^{m}\bar{x_{i}}}{m}$$

$\overset{=}{x}$—The determined value of the candidate whole-wheat flour standard, µg/kg or mg/kg.

$\bar{x_{i}}$—The average value of the determination results of each laboratory µg/kg or mg/kg.

m—Number of laboratories.

For the candidate whole-wheat flour Reference Standards, the identified values have a statement about the measurement uncertainty. According to JJF 1006-1994, assuming that the variables are independent, the combined uncertainty associated with the attribute values of the CRM can be expressed as the following Equation (3)

(3) $$u_{CRM}=\sqrt{u_{char}^{2}+u_{bb}^{2}+u_{lts}^{2}+u_{sts}^{2}}$$

The extended uncertainty is calculated using the following equation, *U_CRM_* = *k* × *u_CRM_*, where *k* is the confidence probability factor. In this CRM, the distribution is assumed to be approximately normal, and the required confidence interval is 95%, *k* = 2.

## 3. Results and Discussion

### 3.1. Preparation of Matrix CRM Candidates

#### 3.1.1. Evaluation of Particle Size Distribution

In the determination of mycotoxins, sample homogeneity was dependent on the particle size distribution of wheat [28]. The homogeneity of DON and ZEN in whole-wheat flour was examined for particle sizes of 250, 500, and 1000 µm, evaluated with a Relative Standard Deviation (RSD).

**Figure 2:**
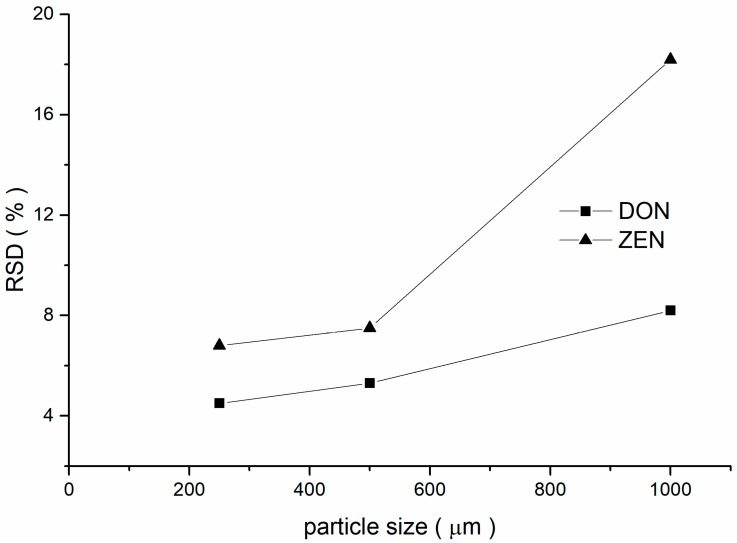
Relative standard deviations of DON and ZEN results at different particle sizes of whole-wheat flour.

**Figure 3:**
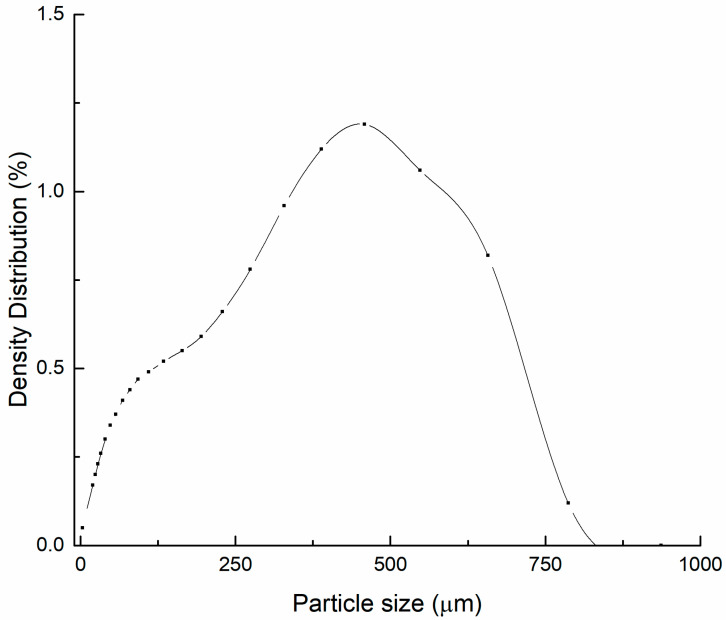
Particle size distribution (Number = 10) of whole-wheat flour.

As the particle size decreased, the RSD% of DON and ZEN content in whole-wheat flour gradually decreased and the uniformity tended to be good (Figure 2). The smaller the particle size of whole-wheat flour, the larger its difference compared to the grain size of daily testing samples. This discrepancy could negatively affect the accuracy of quantity traceability and quality control in daily tests. Through comprehensive consideration, the target particle size for CRMs was 500 μm or less. In total, 30 kg of candidate wheat was homogenized and mixed, then 10 samples were randomly selected and determined using HELOS-OASIS. The particle size of whole-wheat flour less than 374.6 µm accounted for 90% of the total, and less than 560.8 µm accounted for 99%; particle size distributions for CRM are shown in Figure 3. In addition, some long fibres with a length of 500 μm were observed. Fortunately, the homogeneity of the material was not significantly impacted according to the results of the homogeneity test (see Section 3.2).

#### 3.1.2. Study of Homogenizing Time

**Figure 4:**
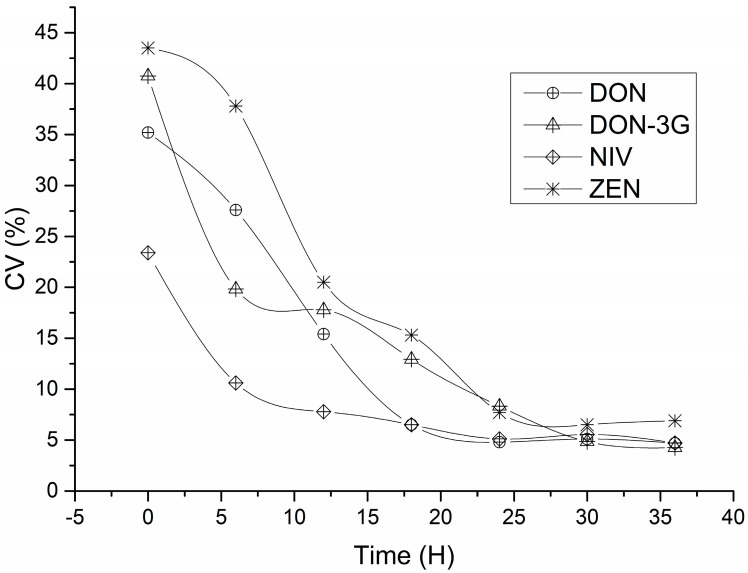
Effect of homogenising time on the homogeneity of *Fusarium* toxin in whole-wheat flour (Number = 6).

Wheat is rich in carbohydrates, proteins, dietary fibre, and trace elements, and its complex matrix makes homogenisation of whole-wheat flour difficult. Therefore, an experiment on mixing time and homogeneity was conducted to prepare the CRM candidate materials. Whole-wheat flour (30 kg) was mixed in a V-mixer at 60 rpm for 36 h. Whole-wheat flour samples were collected from different spatial locations of the mixer at the 6th, 12th, 18th, 24th, 30th and 36th hour of the mixing process at 6 h intervals. Figure 4 shows the relative standard deviations of the measured results for the four *Fusarium* mycotoxins in the whole-wheat flour. The coefficients of variation (CV%) decreased as the mixing time increased. The relative standard deviations of the DON and NIV measurements did not decrease significantly with increasing mixing time beyond 18 h. For DON-3G and ZEN, the relative standard deviations did not decrease significantly beyond a homogenisation time of 30 h. This implies that the homogenisation time should be greater than 36 h for whole-wheat flour. The differences in homogenisation times for different *Fusarium* mycotoxins may be related to the different distribution patterns of these toxins in wheat grains. Studies have shown that DON and NIV are evenly distributed throughout the grain, while ZEN and DON-3G are concentrated in the outer layer of wheat, i.e., the bran. Some studies have shown that the distribution of DON-3G is mainly concentrated in the outer layer of rice bran [29,30,31,32,33].

#### 3.1.3. Effect of Irradiation on the Analyte Mass Fraction

**Figure 5:**
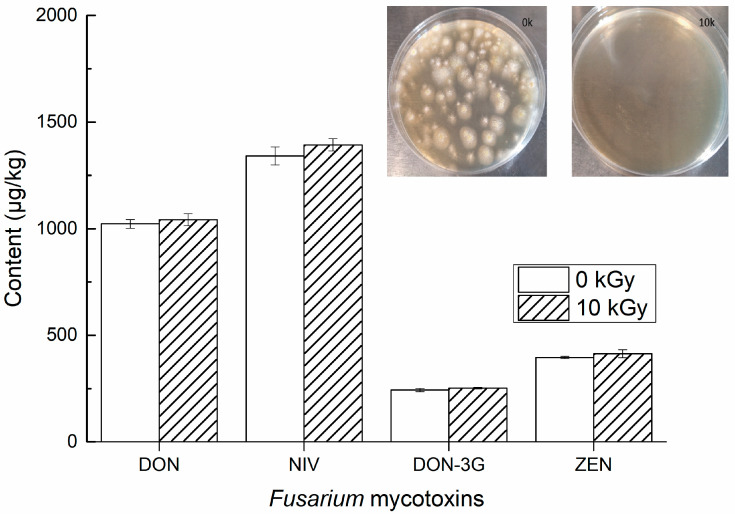
Mycotoxin levels measured using ID-LC-MS/MS in wheat without irradiation (white) after exposure to an absorbed dose of 10 kGy (Stripes). Error bars represent the standard deviation of three replicate sample preparations. The top right image illustrates the impact of varying irradiation doses in reducing the initial microbiological count of whole-wheat flour.

To determine the impact of sterilisation, the levels of incurred mycotoxins were measured using ID-LC-MS/MS in test portions of the incurred wheat before and after exposure to radiation at absorbed doses of 0 and 10 kGy. Figure 5 displays the results acquired from experiments investigating the impact of radiation on the mass proportions of DON, NIV, DON-3G, and ZEN. The results showed that there was no significant difference between the mean values of the two analytes in the irradiated and non-irradiated samples. We also studied the number of vicinal bacteria in whole-wheat flour at different irradiation doses according to the GB 4789.15-2016 [34]. No mould growth was observed at an irradiation dose of 10 kGy. Thus, irradiation treatment at 10 kGy did not alter the concentration of the analytes and could efficiently mitigate the likelihood of *Fusarium* growth and subsequent mycotoxin production during storage. Irradiation treatment of the units is thus an effective way to extend the validity of CRMs.

### 3.2. Homogeneity Testing

**Figure 6:**
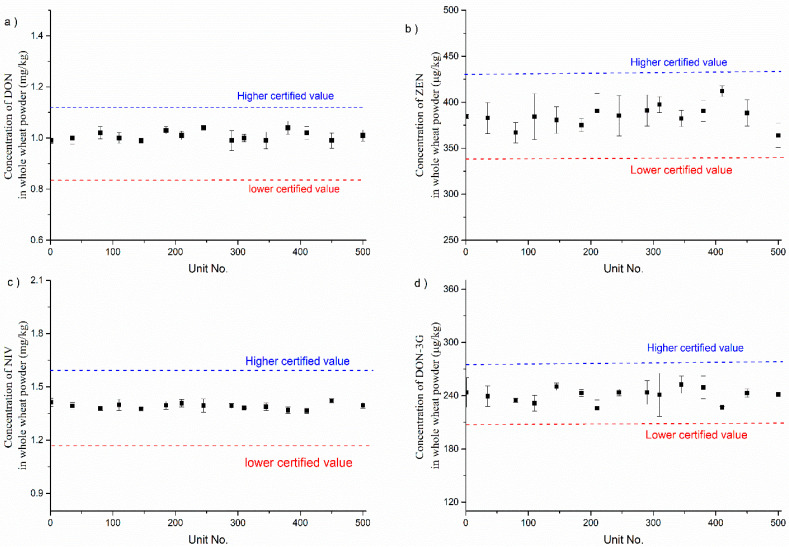
Homogeneity testing results for DON (**a**), NIV (**b**), DON-3G (**c**), and ZEN (**d**) in whole-wheat flour CRM. The error bars indicate the standard deviation within bottles.

Achieving sufficient homogeneity of CRMs is one of our top priorities since mycotoxins are typically found in sporadic mould “hot spots” within grains, and their distribution can be highly variable. However, the homogeneity assessment results suggest that the CRMs prepared in this study effectively addressed any potential issues with the inhomogeneity of CRMs. For homogeneity testing, 15 sample units, randomly selected from the CRM candidates, were measured in triplicate using the ID-LC-MS/MS method. Figure 6 shows the homogeneity test results of the DON, NIV, DON-3G, and ZEN mass fractions in whole-wheat flour within and between bottles. The average concentrations of DON, NIV, DON-3G, and ZEN in the 15 bottles were 1.009 mg/kg, 1.288 mg/kg, 240.6 µg/kg, and 385.0 µg/kg, respectively. According to JJF 1343-2012, we conducted a one-way analysis of variance on the data. The calculated F-values were 1.900, 1.615, 1.671, and 1.499, which were all lower than the critical value of F 0.05 (14, 30) = 2.04. The whole-wheat flour CRM candidates exhibited good homogeneity within and between bottles.

### 3.3. Characterization of CRM Candidates

Standard values of *Fusarium* mycotoxins, such as DON, NIV, DON-3G, and ZEN in whole-wheat flour, were obtained from measurements performed by inter-laboratory comparisons. We organised an inter-laboratory network of six participants. All participants were asked to use the LC-ID-MS/MS method as a reference for two units and three subsamples for each unit. To confirm the accuracy and validity of the measurement results from the participating laboratories, CRMs and blank samples were used for quality control, and the assay results were converted to recoveries for presentation (Table 2). The recoveries of the four *Fusarium* mycotoxins in the six participating laboratories were all between 95 and 105%, indicating that the quality of the CRM can be controlled. The calibrated data were then validated.

The measurement results presented in Table 1 underwent a Shapiro–Wilk test to determine the normal distribution of the mean values (Number = 6) across all laboratories. The resulting *p*-value exceeded 0.05 (95% confidence level), indicating that the average results obtained from different laboratories exhibit normal distribution. The new set of samples (Number = 6) obtained from the mean of the six laboratories underwent the Dixon test (r_1_, r_n_ < ƒ (0.05, 6)) and the Cochrane test (C < C (0.05, 6, 5)). At the 95% confidence level, the statistical tests showed no outliers in these data sets. Therefore, the certified values for the target mycotoxins in whole-wheat flour were DON: 0.983 mg/kg, NIV: 1.370 mg/kg, DON-3G: 241.8 µg/kg, and ZEN: 381.8 µg/kg, which were derived from the mean values of all laboratory means.

### 3.4. Assessment of Stability

**Figure 7:**
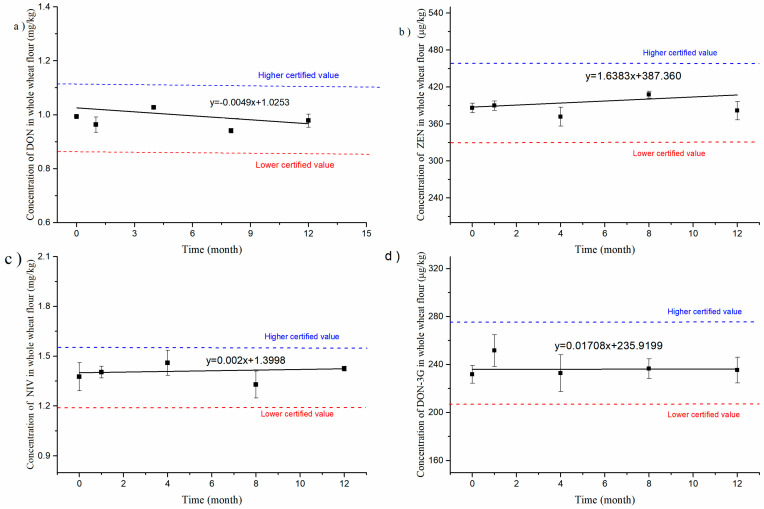
Long-term stability testing results at room temperature for DON (**a**), ZEN (**b**), NIV (**c**), and DON-3G (**d**) in whole-wheat flour CRM.

**Figure 8:**
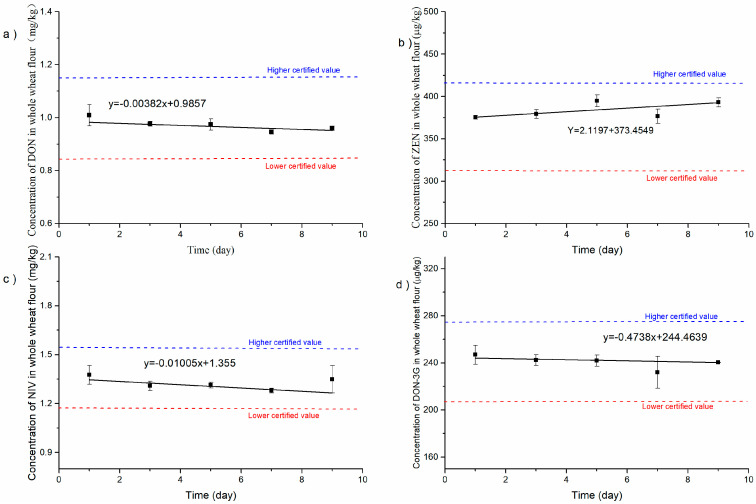
Short-term stability testing results at 40 °C for DON (**a**), ZEN (**b**), NIV (**c**), and DON-3G (**d**) in whole-wheat flour CRM.

For stability testing, DON, NIV, DON-3G, and ZEN in whole-wheat flour CRM candidates have been continuously monitored for 12 months of storage and 9 days of transportation or use according to the classical stability study protocol described in Section 2.6. The results depicting the levels of DON, NIV, DON-3G, and ZEN in whole-wheat flour were plotted against the monitoring time, as illustrated in Figure 7. The data were then statistically analysed using regression analysis. The calculated slope b was not significant for long-term and short-term stability using *t*-tests at the 95% confidence level. This proves that the mycotoxin content in this CRM was stable enough under storage conditions (20 °C). Compared with most grain matrix CRMs stored at −18 °C, the storage condition required for the CRM developed in this laboratory is very user-friendly for storage and use. In addition, the CRM can be successfully transported for nine days under 40 °C without temperature-induced deterioration in mycotoxin contents (Figure 8).

### 3.5. Uncertainty Assessment

**Figure 9:**
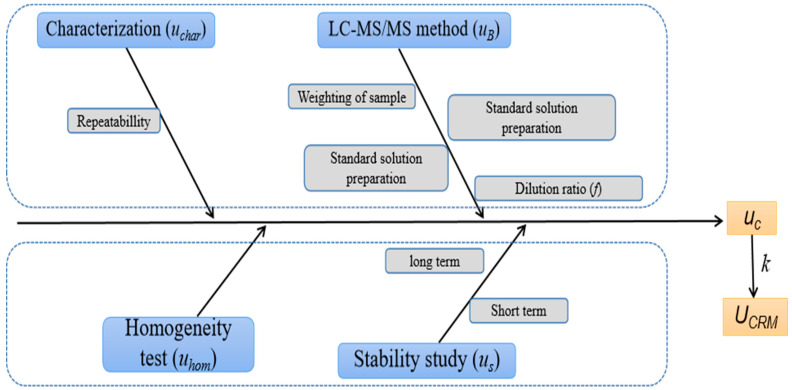
Uncertainty source analysis of whole-wheat flour CRM.

According to Equation (3), the sources of uncertainty for DON, NIV, DON-3G, and ZEN in whole-wheat flour were assignment analysis, homogeneity assessment, long-term stability, and short-term stability monitoring, as shown in the fishbone diagram in Figure 9. The uncertainty of assignment consists of the standard deviation of the mean of multiple laboratory measurements and the uncertainty of measurement model type B (i.e., the concentration of the standard solution, the volume of the sample solution, the mass of the sample, or the concentration of the sample solution). We assigned uncertainties according to the JJF 1059.1-2012 [35]; the sources of uncertainty and the evaluation results are shown in Table 3. Based on these sources, the relative standard uncertainty *u_C_* was calculated. The uncertainty is expressed as the synthetic uncertainty of the assignment, homogeneity, and stability tests with a coverage factor *k* = 2.

### 3.6. Effect of Multiple Reuses on the Moisture of the CRM

**Figure 10:**
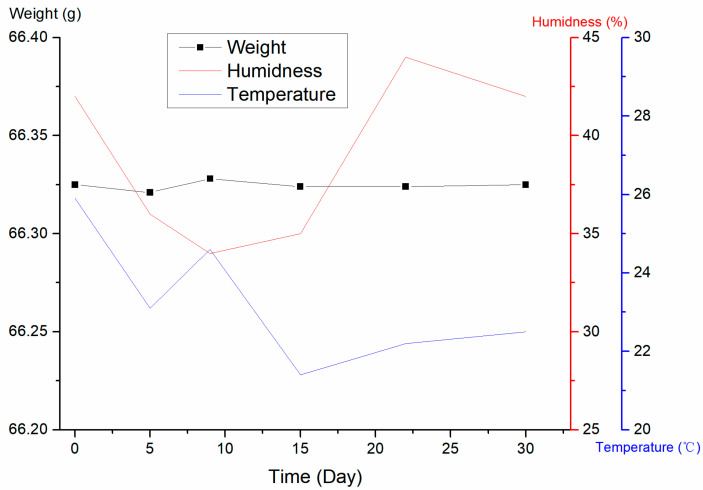
Weight of whole-wheat flour CRM at different temperatures and humidities.

The moisture content of whole-wheat flour was measured using the drying method [36] to be 9.8%, with a relative standard deviation of 1.0% (Number = 9). The CRM was required to be placed at −18 °C for storage after opening the package, which involved multiple freezing and rewarming processes during use. Ambient temperature and humidity may affect the matrix moisture, thus leading to change in the number of mycotoxins. In this study, via simulating the actual use, the bags were placed in the −18 °C refrigerator after opening, removed six times at random times within 30 days, and the whole bags were weighed after rewarming and then stored at −18 °C. The temperature and humidity during the day were recorded, and the results are shown in Figure 10.

The results show that the weight of CRM did not change within 30 days under the ambient temperature of 20–26 °C and a humidity of 32–45%, indicating that no phenomenon, such as moisture absorption, occurred in the laboratory environment. The CRM values can be used directly without dry mass correction.

## 4. Conclusions

In this study, a whole-wheat flour certified reference material (China GBW(E)100816) was developed for the detection of deoxynivalenol and other *Fusarium* mycotoxins. This study included sample homogenisation, analytical methods, characterisation, homogeneity studies (inter-bottle and between-bottle), long- and short-term stability studies, and uncertainty assessment. The whole-wheat flour quality control material naturally contains *Fusarium* mycotoxins, which are deemed more suitable than artificially introduced or microbially fermented cultures. This is because the former shares a physical resemblance with actual samples commonly tested in food laboratories. The amount, homogeneity, and stability of whole-wheat flour were objectively assessed through an ID-LC-MS/MS method established and validated by our laboratory. The CRM showed good homogeneity, and the minimum sample weight was 5 g in the whole-wheat flour CRM. Stability assessments showed that the levels of mycotoxins in the CRM remained stable within their uncertainties for up to one year when stored at room temperature, indicating that freezing is unnecessary. The stability of the CRM will be monitored, and the validity period will be extended based on test results. In addition, the uncertainties arising from the development of CRMs, including identification, homogeneity, and stability, were comprehensively evaluated. The certified values and expanded uncertainties (95% confidence level, *k* = 2) for DON, NIV, DON-3G, and ZEN were determined to be 0.98 ± 0.12 mg/kg, 1.37 ± 0.20 mg/kg, 242 ± 35 μg/g, and 382 ± 50 μg/g. Therefore, this new matrix CRM could be used by testing laboratories for quality control and verification, and will play a crucial role in ensuring the accurate measurement of relevant *Fusarium* mycotoxins in wheat quality and safety monitoring programs.

## Figures and Tables

**Table 1:** MS/MS conditions of DON, NIV, DON-3G and ZEN.

Compounds	Retention Time /min	Ion Mode ^a^	Q1 Mass	Q3 Mass	Declustering Potential/V	Collision Energy/eV	Collision Cell Exit Potential/V
NIV	2.6	−	357.1	281.2 */311.1	−40	−13	10
[^13^C]-NIV	2.6	−	372.1	295.1	−40	−13	10
DON	4.3	+	297.2	249.1 */203.1	55	22/20	7
[^13^C]-DON	4.3	+	312.2	263.1	55	20	7
DON-3G	4.5	+	517.1	427.0 */247.0	50	19/31	14/16
[^13^C]-DON-3G	4.5	+	312.2	245.1	50	31	16
ZEN	14.2	−	317.1	175.0 */130.8	−80	−25/−40	12
[^13^C]-ZEN	14.2	−	335.2	185.0	−80	−41	12

^a^ − negative ion mode; + positive ion mode; * Representative quantitative ion.

**Table 2:** Measurement results of the six participating laboratories.

NO.	DON (mg/kg)			NIV (mg/kg)			DON-3G (µg/kg)			ZEN (µg/kg)		
	Mean	SD	Recovery/%	Mean	SD	Recovery/%	Mean	SD	Recovery/%	Mean	SD	Recovery/%
1	0.989	0.014	94.1	1.297	0.05	99.4	246.8	14.31	102.1	375.6	16.41	102.1
2	1.041	0.015	100	1.468	0.035	103	238.4	7.57	97.8	378.7	14.08	105.3
3	0.863	0.019	93.6	1.342	0.027	98.9	247.2	12.19	96.6	375.1	16.27	100
4	0.974	0.036	97.9	1.465	0.099	96.9	245.2	12.79	102.1	367	16.73	96.8
5	1.017	0.019	100.5	1.302	0.069	95.5	247.8	9.26	96	407.5	19.94	105.4
6	1.011	0.039	97.9	1.348	0.021	95.5	225.2	17.84	103.4	387	16.59	104.5
Overall mean	0.983			1.37			241.8			381.8		
SD	0.063			0.078			8.82			14.14		

SD, standard deviation.

**Table 3:** Uncertainty budget for the certified CRM values.

Source of Uncertainty	Relative Uncertainty (%)			
	DON	NIV	DON-3G	ZEN
Characterization by the co-laboratory study (*u_char_*)	2.66	2.31	1.49	1.51
Method (*u_B_*)	2.22	1.78	2.89	1.72
Inhomogeneity (*u_hom_*)	1.19	0.71	2.08	1.74
Instability (*u_s_*)	4.83	6.58	5.28	5.75
Combined standard uncertainty (*u_c_*)	6.06	7.23	7.18	6.43

## Data Availability

The data used to support the findings of this study can be made available by the corresponding author upon request.
